# Training Status as a Marker of the Relationship between Nitric Oxide, Oxidative Stress, and Blood Pressure in Older Adult Women

**DOI:** 10.1155/2016/8262383

**Published:** 2015-12-01

**Authors:** André Mourão Jacomini, Hugo Celso Dutra de Souza, Danielle da Silva Dias, Janaina de Oliveira Brito, Lucas Cezar Pinheiro, Anderson Bernardino da Silva, Roberta Fernanda da Silva, Atila Alexandre Trapé, Kátia De Angelis, José Eduardo Tanus-Santos, Sandra Lia do Amaral, Anderson Saranz Zago

**Affiliations:** ^1^Ribeirao Preto Medical School, University of Sao Paulo (USP), Avenida Bandeirantes 3900, 14049-900 Ribeirao Preto, SP, Brazil; ^2^Translational Physiology Laboratory, Universidade Nove de Julho (UNINOVE), Rua Vergueiro 235/249, Liberdade, 01504-001 Sao Paulo, SP, Brazil; ^3^School of Nursing of Ribeirao Preto, University of Sao Paulo (USP), Avenida Bandeirantes 3900, 14040-902 Ribeirao Preto, SP, Brazil; ^4^Department of Physical Education, Universidade Estadual Paulista (UNESP), Avenida Engenheiro Luiz Edmundo Carrijo Coube, 14-01, Bairro Vargem Limpa, 17033-360 Bauru, SP, Brazil

## Abstract

The purpose of this study was to evaluate the influence of functional fitness and oxidative capacity on the nitric oxide concentration associated with hemodynamic control in older adult women. The sample consisted of 134 women (65.73 ± 6.14 years old). All subjects underwent a physical examination to assess body mass index, waist-hip ratio, body fat measurement by dual energy X-ray absorptiometry, and blood pressure (BP). Training status (TS) was evaluated by indirect determination of maximal oxygen uptake by a treadmill test using Balke protocol modified for older adults. Functional fitness was also evaluated through a “Functional Fitness Battery Test” to determine the general fitness functional index (GFFI). All participants were separated according to the functional fitness (TS1, very weak and weak; TS2, regular; TS3, good and very good). Plasma blood samples were used to evaluate prooxidant and antioxidant activity and nitrite and nitrate concentrations. The general results of this study showed that good levels of TS were related to lower levels of lipoperoxidation and protein damage, higher levels of antioxidant, and higher concentration of nitrite and nitrate. This combination may be responsible for the lower levels of BP in subjects with better TS.

## 1. Introduction

Considered as a chronic-degenerative disease, hypertension (HT) has a high incidence among elderly people and has several factors involved in its etiology, such as environmental, genetic, psychological, and humoral factors [1, 2]. Among them, low concentration of nitric oxide (NO) and high exposure to oxidative stress (OS) have been receiving special attention due to the impairment in the vasodilatation mechanism which can compromise the normal values of blood pressure (BP).

Nitric oxide is produced by endothelial cells and is being considered as a potent vasodilator due to the effects in the vascular smooth muscle relaxation [3]. So, low concentration of NO is associated with increased values of BP, especially in older people [4, 5].

Oxidative stress is also considered in the HT etiology because it affects many functions in the organism. Actually, reactive oxygen species (ROS), such as superoxide anions (O_2_^−·^), hydroxyl radical (^·^OH), and hydrogen peroxide (H_2_O_2_), are physiologically produced in low concentrations and work as a molecular signaling to maintain vascular integrity and as a regulator of endothelial function [6]. However, in high concentration, O_2_^−·^ can induce a deleterious effect in the organism [7]. For example, the interaction between O_2_^−·^ and NO has an important role in vascular homeostasis [8] because of the formation of peroxynitrite molecules (ONOO−) [9, 10], which are largely responsible for the process of endothelial dysfunction and decrease of NO bioavailability [6, 11].

In short, both mechanisms can compromise the vasodilatation, resulting in elevated values of BP. However, it has been shown that regular physical exercise has an important function against these deleterious effects. The main mechanism for that is related to shear stress by increased blood flow during the physical activity [12, 13]. This in turn contributes to improving endothelial function due to the stimulus for the production of NO and the endothelial superoxide dismutase (ecSOD), an antioxidant that has a high affinity with O_2_^−·^, counteracting the reaction between NO and O_2_^−·^, consequently increasing the NO bioavailability [6, 10, 14–16].

Although many studies have been demonstrating that physical exercise promotes an improvement in NO concentration by increased endothelial nitric oxide synthase (eNOS) activity and oxidative stress [17, 18] through the increased stimulus of superoxide dismutase (SOD), catalase (CAT), and glutathione peroxidase (GPx) [16, 19], the effectiveness of physical exercise is still controversial. For example, Trapé et al. demonstrated that good level of training status improved the levels of nitrite concentration but with no change in TBARS [20]. Gomes et al. [21] found decreased levels in malondialdehyde (MDA) and an increased level of whole blood nitrite concentration but no difference in plasma nitrite after 3 months of physical exercise in patients with metabolic syndrome. And finally, Bouzid et al. compared young and elderly people and showed that some oxidative stress markers (SOD, GPx, and glutathione reductase) improved after acute exercise only in the young groups and just the MDA increased in the elderly group [22]. These results demonstrated low antioxidant efficiency with aging.

All these differences can be mainly due to the differences among intensity, type, duration, and frequency in which physical exercise can be practiced, especially those related to elderly people [23–25]. The study of Santana et al. confirms this statement. The authors evaluated the plasma nitrite in hypertensive older women after the peak of incremental test and after 90% of anaerobic threshold acute exercise and only in the incremental test the values of nitrite increased. These results showed that there is a difference in nitrite concentration according to the intensity of exercise [26].

Independent of the physical exercise practiced, exercise should be good enough to raise fitness level of the practitioner, thus bringing the benefits previously described. However, there are still few studies seeking the establishment of the relationship through the current TS of the practitioner and the benefits of the practice. With this background the hypothesis of this study was that elderly people with good level of TS, regardless of the type of exercise performed, will present better relationship between BP, NO concentration, and OS. Therefore, the purpose of this study was to investigate if the relationship between BP, NO, and OS can be modulated by the TS in older adult women.

## 2. Methods

### 2.1. Sample Selection

All procedures were previously approved by Institutional Review Board of University of São Paulo State (CEP/FC-UNESP n° 323.427) and all subjects provided written consent before the beginning of experiments. Extension programs linked to universities and associations of retirees related to the elderly community were visited and all individuals were invited to participate in this study with the same chance to be included once they met the inclusion criteria.

### 2.2. Inclusion and Exclusion Criteria

Subjects should be nonsmoking, nonalcoholic, and nondiabetic, should be within the age of 50–80 years, should not have cardiovascular peripheral, cerebrovascular, neurologic, or psychiatric diseases (angina, vascular disease, etc.), should not have maximal systolic blood pressure (SBP) > 160 mmHg and maximal diastolic blood pressure (DBP) > 100 mmHg or other health conditions which could compromise the achievement of motor tests, and should have normal values of lipid and glycemic profile. The subject medical history was reviewed on their first visit.

### 2.3. Blood Collection

Plasma samples were prepared from whole blood samples obtained from fasting individuals by venous puncture with heparinized vials for further analysis of oxidant and antioxidant profile and nitrite and nitrate concentration. All participants were informed to avoid foods rich in nitrate (beet, cabbage, spinach, and lettuce) in the last day before the blood collection.

#### 2.3.1. Protein Assay

Proteins were quantified by the method of Lowry et al., which uses a bovine albumin solution in the concentration of 1 mg/mL as standard, using 10 *μ*L of sample. Such quantification was used to correct the calculation of the following analysis: SOD, TBARS, and oxidatively modified proteins [27].

#### 2.3.2. Thiobarbituric Acid Reactive Substances (TBARS)

Plasma lipid peroxide levels were determined by measuring TBARS, a common method for measuring the concentration of malondialdehyde, the main breakdown product of oxidized lipids. For the TBARS assay, using 250 *μ*L of sample, trichloroacetic acid (10%, w/v) was added to the homogenate to precipitate proteins and to acidify the samples. This mixture was then centrifuged (4000 rpm, 10 min), the protein-free sample was extracted, and thiobarbituric acid (0.67%, w/v) was added to the reaction medium. The tubes were placed in a water bath (100°C) for 30 min. The absorbencies were measured at 535 nm using a spectrophotometer [28].

#### 2.3.3. Oxidatively Modified Proteins

The protein damage was determined by protein carbonyls measurements, using 200 *μ*L of sample. Plasma samples were incubated with 2,4-dinitrophenylhydrazine (DNPH 10 mM) in a 2.5 M HCl solution for 1 h at room temperature in the dark. Samples were vortexed every 15 min. Subsequently, a 20% trichloroacetic acid (w/v) solution was added and the solution was incubated on ice for 10 min and centrifuged for 5 min at 1000 g to collect protein precipitates. An additional wash was performed with 10% trichloroacetic acid (w/v). The pellet was washed three times with ethanol/ethyl acetate (1 : 1) (v/v). The final precipitates were dissolved in 6 M guanidine hydrochloride solution and incubated for 10 min at 37°C, and the absorbance was measured at 360 nm [29].

#### 2.3.4. Nitrite (**N** **O**_2_^−^) Concentrations

The nitrite content of the samples was analyzed using an ozone-based reductive chemiluminescence assay. For this, 50 *μ*L of plasma samples was injected into a solution of acidified triiodide, purging with nitrogen in line with a gas-phase chemiluminescence NO analyzer (Sievers Model 280 NO Analyzer, Sievers, Boulder, CO, USA). Approximately 8 mL of triiodide solution (2 g of potassium iodide and 1.3 g of iodine dissolved in 40 mL of water with 140 mL of acetic acid) was placed in the purge vessel into which plasma samples were injected. The data were analyzed using the software Origin Lab 6.1 [30].

#### 2.3.5. Nitrate (**N** **O**_3_^−^) Concentrations

To determine the nitrate concentration, 40 *μ*L of plasma was incubated with the same volume of nitrate reductase buffer (0.1 M potassium phosphate, pH 7.5, containing 1 mM *β*-nicotinamide adenine dinucleotide phosphate and 2 Uf nitrate reductase/mL) in individual wells of a 96-well plate. Samples were allowed to incubate over night at 37°C in the dark. Eighty microliters of freshly prepared Griess reagent (1% sulfanilamide and 0.1% naphthyl ethylenediamine dihydrochloride in 5% phosphoric acid) was added to each well and the plate was incubated for an additional 15 min at room temperature. A standard nitrate curve was obtained by incubating sodium nitrate (0.2–200 mM) with the same reductase buffer [30].

#### 2.3.6. Measurement of SOD Activity

The technique used to measure SOD activity is based on inhibition of the superoxide radical reaction by pyrogallol, a compound that oxidizes itself with the pH variation, leading to formation of a colored product, detected spectrophotometrically at 420 nm for 2 minutes. The percentage of inhibition of initial reaction rates depends on the pH and the quantity of SOD present in the reaction mixtures. Thus, the quantity of enzyme required to inhibit the reaction by 50% is defined as one unit of SOD. 968 *μ*L of Tris-phosphate buffer was added to 50 mM/L (pH 8.2), 8 *μ*L of pyrogallol to 24 mM/L, and 4 *μ*L CAT 30 mM/L in 20 *μ*L of samples. Three different concentrations of SOD (0.25 U, 0.5 U, and 1 U) were used to make the standard curve, which provided an equation of the line for purposes of the calculation. Thus, the SOD activity was determined by measuring the rate of formation of the oxidized pyrogallol [31].

#### 2.3.7. Measurement of GPx Activity

The GPx activity was measured by reaction with glutathione reductase, measuring the consumption of NADPH in the reduction reaction coupled to the GPx reaction, using an enzymatic-colorimetric method by commercial kit (Cayman Chemical Company, Ann Arbor, MI, USA).

### 2.4. Blood Pressure (BP)

Blood pressure was measured after 5 min of rest on three separate days according to the VI Brazilian Hypertension Guidelines [32], using an adequate aneroid sphygmomanometer to the circumference of the arm and a stethoscope placed over the brachial artery.

### 2.5. Physical Examination

All subjects underwent a physical examination to assess training status (TS), body composition, and anthropometric measurements. Participants were also questioned about the current habits of physical exercise. The baseline testing was performed at least 24 h after the last exercise session.

#### 2.5.1. Training Status (TS)

Indirect determination of maximal oxygen uptake ($\dot{V}O_{2}max$) was performed by a treadmill test using Balke protocol modified for older adults [33], which allowed an estimation of participant's $\dot{V}O_{2}max$. The “Battery Test” proposed by the “American Alliance for Health, Physical Education, Recreation and Dance” (AAHPERD) was also used to assess the functional fitness of the participants, evaluating the following physical capabilities: coordination, flexibility, muscular strength and endurance, dynamic agility, and cardiovascular endurance as previously described [34–36]. The sum of the percentiles of each test was used to calculate the GFFI. All participants were divided according to the TS (TS1, very weak and weak GFFI: 0 to 199 points; TS2, regular GFFI: 200 to 299 points; TS3, good and very good GFFI: 300 to 500 points). Due to low frequency of participants in the groups “very weak” and “very good,” the GFFI classification led to adjustments as the division of groups. Respecting the minimum of 10% of participants in each group to perform the statistical analysis [37], it was decided to unite the groups “very weak” and “weak” and unite the groups “good” and “very good.”

#### 2.5.2. Body Composition

Body composition was measured by dual energy X-ray absorptiometry scan (DEXA, Discovery Wi/HOLOGIC Inc., Bedford, USA). The equipment was calibrated before the data collection, according to the manual instructions. After completion of the scans, the body composition was analyzed using the software settings, thus providing the results of total body fat mass, android and gynoid fat mass, and bone mineral density.

#### 2.5.3. Anthropometric Indicators

After anthropometric measurement of waist and hip circumference, body weight, and height, the waist-hip ratio was calculated dividing the waist circumference (cm) by the hip circumference (cm), while body mass index (BMI) was calculated dividing body weight (kg) by the square of the height (m).

### 2.6. Statistical Analysis

Descriptive statistics was calculated and Pearson's correlation coefficient was performed to detect correlation among variables. One-way ANOVA with Tukey's post hoc test was performed to assess statistically significant differences between groups (*p* < 0.05). TS was considered as independent variable. The data were analyzed by SPSS 17.0 statistical package. Power analysis was calculated using the prevalence of 30% of hypertension in the Brazilian population and 80% of power and significance level of 5% [32].

## 3. Results

**Figure 1:**
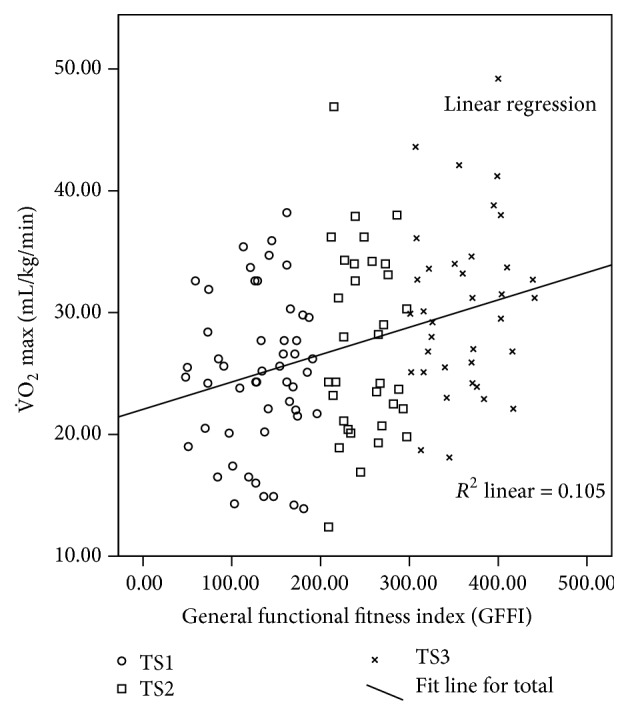
Pearson correlation coefficient between general functional fitness index (GFFI) and $\dot{V}O_{2}max$.  ^*∗*^Correlation is significant at the 0.01 level.

From all clusters visited, 134 older adult women (65.75 ± 6.14 years old) met our initial inclusion criteria.  Figure 1 presents the correlation between $\dot{V}O_{2}max$ and GFFI. It can be observed that good levels in the GFFI were associated with high values of $\dot{V}O_{2}max$. Even though both variables have a moderate correlation, the authors opted to use the GFFI as independent variable, because the GFFI comprises a multicomponent assessment which is in agreement with American College of Sports Medicine Guidelines for the elderly [24].

Subjects' characteristics are shown in Table 1. TS3 was 3 years younger than other groups. For anthropometrics variables, android fat mass was lower in TS2 and bone mineral density was lower in TS3 compared to the other respective groups. Despite these differences, the sample of this study was considered homogeneous for body composition parameters.

About the TS, as expected, TS3 presented higher values of GFFI and $\dot{V}O_{2}max$ compared to the other groups (Table 1). These results are in accordance with the number of physical exercises that the participant performed.

**Figure 2:**
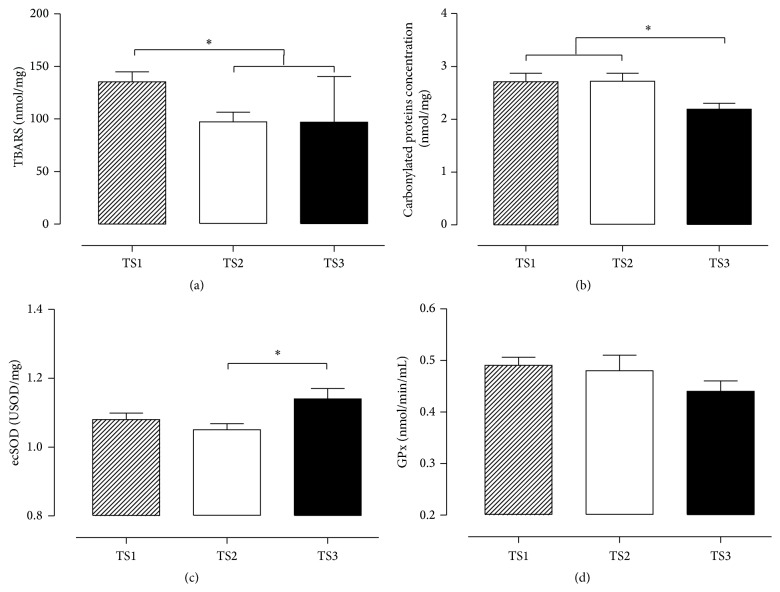
Lipoperoxidation (TBARS) values expressed in nmol/mg protein (a); protein oxidation (carbonyls) values expressed in nmol/mg protein (b); ecSOD activity values expressed in USOD/mg protein (c); GPx activity values expressed in nmol/min/mL (d); TS1, very weak and weak GFFI; TS2, regular GFFI; TS3, good and very good GFFI. ^*∗*^ *p* < 0.05.

Figure 2 demonstrates the oxidative profile between groups. The lipoperoxidation (TBARS) was lower in TS3 and TS2 compared to TS1 (a), and lower damage to protein was observed in TS3 (b). For antioxidant capacity, higher SOD activity was observed in TS3 compared to TS2 (c). However, no difference was found in the GPx activity between groups (d).

**Figure 3:**
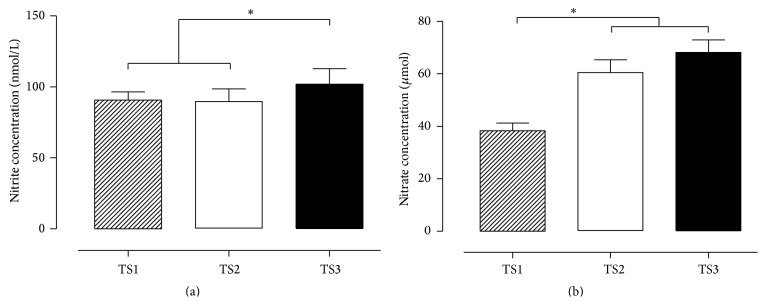
Nitrite values expressed in nmol/L (a); nitrate values expressed in *μ*mol (b); TS1, very weak and weak GFFI; TS2, regular GFFI; TS3, good and very good GFFI. ^*∗*^ *p* < 0.05.

The results presented in Figure 3 demonstrate that individuals with better TS (TS3) have higher plasma nitrite concentrations compared to TS1 and TS2 groups (a), while TS2 and TS3 show higher values of nitrate concentrations compared to TS1 (b).

**Figure 4:**
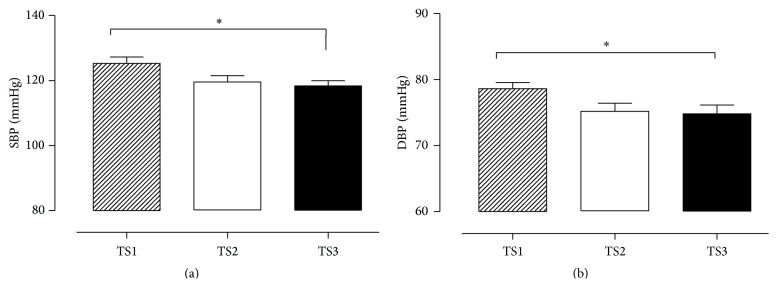
Systolic blood pressure (SBP, (a)) and diastolic blood pressure (DBP, (b)) values expressed in mmHg; TS1, very weak and weak GFFI; TS2, regular GFFI; TS3, good and very good GFFI. ^*∗*^ *p* < 0.05.

For hemodynamic variables, it can be observed that TS3 presented lower values of SBP and DBP compared with TS1 (Figure 4). There was no difference between groups about the use of the number of antihypertensive drugs (TS1 = 0.72 ± 1.07; TS2 = 0.77 ± 0.83; TS3 = 0.75 ± 0.92).

## 4. Discussion

With the purpose to evaluate if the relationship between BP, NO, and OS can be modulated by TS in older adult women, the general results of this study showed that good levels of TS were related to lower levels of ROS damage (TBARS and protein carbonyls, responsible for measuring membrane and protein damage), higher levels of antioxidant (ecSOD), and higher concentration of nitrite and nitrate. This combination was related to the lower levels of BP in older adult women with good level of training status.

GFFI was chosen to represent the training status of the participants although the correlation between GFFI and $\dot{V}O_{2}max$ was moderate (*r* = 0.324; *p* < 0.01); however previous study of our laboratory demonstrated a good correlation between GFFI and $\dot{V}O_{2}max$ [20]. In addition, the GFFI represents a multicomponent assessment which is in agreement with ACSM guidelines [24].

Regarding OS profile, our results showed that subjects with good level of TS demonstrated lower prooxidant profile and higher antioxidant profile. These results are in accordance with Traustadottir et al., who compared physically fit older adults, men and women, with sedentary age-matched controls, demonstrating that greater physical fitness is associated with lower oxidative stress and greater antioxidant capacity in older adults [33].

Physical exercise is the main stimulus to supply the energy demand required to perform certain activity. Krebs cycle and electron transport chain are stimulated for ATP resynthesis, resulting in a marked increase of ROS, especially O_2_^−·^. However, physical exercise also activates some genes responsible for the generation of antioxidant components, such as SOD, CAT, and GPx, as described previously [17, 18].

Together, these results take an important function for blood pressure control due to the increased vasodilatation mechanism. Although there are several conditions that can contribute to endothelial dysfunction, OS levels and aging are involved in hypertension, both leading to an increased production of ROS. Judge et al. compared young and old male Fischer-344 rats and observed a significant increase in OS levels in older animals, which showed a higher concentration of H_2_O_2_, protein carbonyls, and TBARS and also a significant age-related increases in MnSOD, GPx, and CAT activities [34]. However, in the current study, TS3 was associated with lower OS effect which was associated with higher NO concentration. Considering that the reactions between NO and superoxide anion are most likely a major cause of impaired endothelium dependent vasorelaxation in hypertension [35, 36], the reduction in OS may be responsible for increase in NO bioavailability.

The SOD is one of the major defense systems for removal of O_2_^−·^, acting as a catalyst for the dismutation of O_2_^−·^ (O_2_^−·^ + O_2_^−·^ + 2H^+^ → O_2_ + H_2_O_2_), while GPx and CAT play an important role in the elimination of hydrogen peroxide (H_2_O_2_), promoting its catalysis by water, avoiding the accumulation of O_2_^−·^ and H_2_O_2_, so there is no production of hydroxyl radical, against which there is an enzymatic defense system [37–39]. A lower activity of SOD and GPx enzymes has been observed, besides a high production of H_2_O_2_ in hypertensive subjects newly diagnosed and untreated, being inversely correlated to BP [40].

The study of Bouzid et al. divided older adults according to their TS level based on answer to a physical activity questionnaire and observed that, during postexercise period, antioxidant activity (ecSOD, GPx, and *α*-tocopherol) increased in both groups (low and high fitness level), but it was not observed in the sedentary group [41].

Although no difference was found in the GPx activity between groups, the current study showed that better levels of TS (TS3) were associated with higher SOD activity and NO concentration, thus suggesting higher NO bioavailability. According to this finding, a previous study of Trapé et al. also showed that nitrite concentration was higher in better TS group, although there was no difference for TBARS [20]. Additionally, the study of Vasconcelos et al. showed that hypertensive patients showed higher oxidative damage and lower antioxidant markers, suggesting that they were under oxidative stress [42]. Thus, this relationship seems to be responsible for the better BP control.

Besides the fact that all participants have taken the same number of antihypertensive drugs and all values of BP had been considered normal, TS3 presented low values of BP in this study.

Thereby, physical exercise has been related to increased eNOS activity and antioxidant system due to stimulus of peroxisome proliferator-activated receptor gamma coactivator 1-alpha (PGC-1*α*). By modulating the ratio of ATP/ADP, which is essential to maximize the energy associated with muscular contraction [43], physical exercise has an important function in the regulation of enzyme adenosine monophosphate kinase (AMPK) activity [44–47]. The activation of AMPK increases the expression of PGC-1*α* in skeletal muscle [18, 48, 49], which induces the expression of lipid catabolism genes (*β*-oxidation), electron transport chain [17, 18, 50], and activation of some genes responsible for generation of antioxidant components such as superoxide dismutase (SOD), catalase (CAT), and glutathione peroxidase (GPx) [17, 18]. Thus, increased expression of PGC-1*α* by physical exercise is associated with increased antioxidant enzyme activity, thereby opposing the OS.

## 5. Conclusions

In general, the results of the present study suggest that the relationship between BP, NO, and OS can be modulated by the TS because better results in the TS are associated with lower ROS molecular damage and higher NO bioavailability, both contributing to an improved blood pressure control in older adult women.

It is important to note that the novelty aspect of this study is the way of assessment of fitness. The physical activity can be practiced by different forms promoting different adaptations in human body. Adding that to the ACSM recommendations for multicomponent exercises for elderly people, the general training status seems more adequate to establish the relationship with health. However this statement still has not been established by the literature, as was demonstrated by the current study.

## 6. Limitations of the Study

The authors understand that ambulatory BP measurements are more accurate than office BP measurements. However, we did not have this technique available at the time of data collection. No specific control of diet was performed; however, participants were requested to have a light meal before the test. The hormonal therapy was not controlled in this study. Although it can be considered one of the factors that affect the endothelial function by altering degradation of NO, the information in the anamneses questionnaire was not enough to be included in this study. The use of antihypertensive drugs was not interrupted during the study.

## Figures and Tables

**Table 1:** Subjects' characteristics.

Variables	TS1 (*n* = 54)	TS2 (*n* = 40)	TS3 (*n* = 40)	Ranges of all participants	
				Minimum	Maximum
Age (years)	66.85 ± 5.89	66.47 ± 5.31	63.47 ± 6.76^a^	50.00	79.00
Anthropometry and body composition variables					
Body mass index (kg/m^2^)	29.46 ± 5.11	27.22 ± 6.75	27.23 ± 3.83	19.10	35.00
Waist-hip ratio	0.89 ± 0.09	0.88 ± 0.06	0.89 ± 0.08	0.34	1.33
Total body fat mass (%)	42.34 ± 5.07	40.53 ± 5.84	39.71 ± 4.59	28.20	51.95
Android fat mass (%)	43.59 ± 6,44	39.66 ± 7.68^a^	40.68 ± 6.43	20,72	51.77
Gynoid fat mass (%)	42.92 ± 4.65	41.42 ± 5.81	41,30 ± 5.74	25.9	51.07
Bone mineral density (g/cm^2^)	0.86 ± 0.10	0.84 ± 0.07	0.79 ± 0.09^a^	0.57	1.10
Functional fitness variables					
Coordination (s)	16.35 ± 4.71	12.26 ± 2.29^a^	10.59 ± 1.74^a^	8.30	37.16
Flexibility (cm)	51.19 ± 10.91	58.24 ± 10.59^a^	61.76 ± 9.23^a^	21.00	80.5
Muscular strength (rep)	18.96 ± 4.05	23.77 ± 3.77^a^	27.35 ± 3.53^ab^	10.00	37.0
Agility (s)	31.54 ± 4.68	24.32 ± 2.69^a^	20.52 ± 2.32^ab^	16.17	45.77
Endurance (s)	584.42 ± 76.39	533.73 ± 52.96^a^	473.05 ± 35.02^ab^	355.00	802.00
GFFI (score)	130.47 ± 42.73	249.40 ± 28.25^a^	361.50 ± 40.21^ab^	35	441
$\dot{V}O_{2}max$ (mL/kg/min)	24.65 ± 6.85	27.54 ± 7.78	30.94 ± 7.46^a^	12.4	49.20
Number of exercises performed	1.24 ± 0.82	1.50 ± 0.81	1.70 ± 1.06^a^	0	5

GFFI, general functional fitness index; TS1, very weak and weak GFFI; TS2, regular GFFI; TS3, good and very good GFFI. Values are mean (SE).

^a^ *p* < 0.05 versus TS1.

^b^ *p* < 0.05 versus TS2.
